# Machine learning-based myocardial infarction bibliometric analysis

**DOI:** 10.3389/fmed.2025.1477351

**Published:** 2025-02-06

**Authors:** Ying Fang, Yuedi Wu, Lijuan Gao

**Affiliations:** Xiaoshan District Hospital of Traditional Chinese Medicine, Hangzhou, Zhejiang Province, China

**Keywords:** machine learning, myocardial infarction, bibliometrics, CiteSpace, deep learning

## Abstract

**Purpose:**

This study analyzed the research trends in machine learning (ML) pertaining to myocardial infarction (MI) from 2008 to 2024, aiming to identify emerging trends and hotspots in the field, providing insights into the future directions of research and development in ML for MI. Additionally, it compared the contributions of various countries, authors, and agencies to the field of ML research focused on MI.

**Method:**

A total of 1,036 publications were collected from the Web of Science Core Collection database. CiteSpace 6.3.R1, Bibliometrix, and VOSviewer were utilized to analyze bibliometric characteristics, determining the number of publications, countries, institutions, authors, keywords, and cited authors, documents, and journals in popular scientific fields. CiteSpace was used for temporal trend analysis, Bibliometrix for quantitative country and institutional analysis, and VOSviewer for visualization of collaboration networks.

**Results:**

Since the emergence of research literature on medical imaging and machine learning (ML) in 2008, interest in this field has grown rapidly, particularly since the pivotal moment in 2016. The ML and MI domains, represented by China and the United States, have experienced swift development in research after 2015, albeit with the United States significantly outperforming China in research quality (as evidenced by the higher impact factors of journals and citation counts of publications from the United States). Institutional collaborations have formed, notably between Harvard Medical School in the United States and Capital Medical University in China, highlighting the need for enhanced cooperation among domestic and international institutions. In the realm of MI and ML research, cooperative teams led by figures such as Dey, Damini, and Berman, Daniel S. in the United States have emerged, indicating that Chinese scholars should strengthen their collaborations and focus on both qualitative and quantitative development. The overall direction of MI and ML research trends toward Medicine, Medical Sciences, Molecular Biology, and Genetics. In particular, publications in “Circulation” and “Computers in Biology and Medicine” from the United States hold prominent positions in this study.

**Conclusion:**

This paper presents a comprehensive exploration of the research hotspots, trends, and future directions in the field of MI and ML over the past two decades. The analysis reveals that deep learning is an emerging research direction in MI, with neural networks playing a crucial role in early diagnosis, risk assessment, and rehabilitation therapy.

## 1 Introduction

Myocardial infarction (MI) is a life-threatening coronary-associated pathology, characterized by sudden cardiac death (1). It represents a serious and potentially fatal condition, being the leading cause of death worldwide and affecting 32.4 million people annually (2). Adverse vascular events occur in 20–40% of acute myocardial infarction (AMI) cases within 2 years of onset. These events significantly contribute to the high mortality rates associated with MI. Identifying high-risk clinical predictors and enhancing the management of at-risk patients can improve the long-term prognosis of patients, potentially leading to a reduction in the incidence of major adverse cardiovascular events (MACE) such as recurrent MI, stroke, and heart failure (3).

However, in the early stages of AMI, cardiac enzymes may not be elevated, and ECG signals reflecting cardiac electrical activity exhibit transient morphological changes during the process of myocardial ischemia caused by MI. This situation can only be detected through thorough initial investigations (4). According to the ‘China Cardiovascular Health and Disease Report 2021,’ the mortality rate of AMI increased by 3.5 times in rural areas and 2.66 times in urban areas from 2002 to 2019. In 2019, the AMI mortality rate was 0.08% in rural areas and 0.06% in urban areas. Furthermore, recent studies have highlighted significant disparities in the care and outcomes of MI patients across hospitals with varying levels of care. Swift diagnosis and timely reperfusion therapy play a pivotal role in reducing mortality associated with myocardial infarction (5).

Machine learning (ML) encompasses a suite of advanced techniques capable of automatically discerning patterns within data and leveraging these patterns to forecast future outcomes or make decisions amid uncertainty. Specifically, ML algorithms can analyze large volumes of patient data, including electrocardiogram (ECG) readings, clinical history, and biomarker levels, to detect subtle signs of MI that may be missed by traditional methods. By employing ML methods, rapid and precise predictions can be achieved, facilitating the expedited diagnosis of acute myocardial infarction (AMI) in emergency cardiology settings (3).

Recently, bibliometric analyses of ML have gained prominence across diverse research domains, including stroke (6), cancer (7), pulmonary artery hypertension (8), cardiovascular surgery (9), and arrhythmia (10). Hassannataj Joloudari et al. have comprehensively reviewed the application of artificial intelligence (AI) technology in the automated detection of myocardial infarction (11). In particular, despite the burgeoning literature on ML, existing bibliometric studies have yet to delve into the specific intersection of ML and myocardial infarction in depth.

Bibliometric analysis is a methodology that integrates mathematics, statistical methods, and data visualization technology to comprehensively evaluate research outcomes. CiteSpace is literature visualization software created by Professor Chen Chaomei from the Department of Information Science at the School of Computer and Information Science, Drexel University, USA (12, 13). Bibliometrix is bibliometric software programmed in R (14). VOSviewer is bibliometric software with enhanced visualization capabilities, designed for constructing and viewing bibliometric maps. It can be used to build maps of authors, journals, or keywords based on co-occurrence data (15, 16).

This study delves into the evolutionary trajectory of machine learning (ML) and myocardial infarction (MI) research over the past two decades. It marks the inaugural endeavor to leverage bibliometric analysis in scrutinizing publications pertaining to ML and MI, aiming to garner insights into the global trends in harnessing ML for myocardial infarction research.

## 2 Materials and methods

### 2.1 Data retrieval strategy

We conducted a comprehensive retrospective bibliometric analysis of journal articles pertaining to myocardial infarction (MI) and machine learning (ML) published between 1 January 2004 and 31 December 2023. This time frame encompasses the emergence and rapid expansion of ML research in the field of MI, enabling us to perform a comprehensive examination of trends and hotspots. To gather our dataset, we employed the Web of Science Core Collection database, leveraging a meticulously designed search strategy.

Search Strategy: To compile a comprehensive dataset, we devised a meticulous search strategy utilizing the Web of Science Core Collection database. The search terms encompassed the primary concepts of “myocardial infarction” in conjunction with a range of machine learning methodologies, specifically “machine learning,” as well as various advanced techniques such as “Naive Bayes,” “decision trees,” “random forest,” “support vector machines,” “gradient boosting decision tree,” “adaptive boosting,” “extreme gradient boosting,” “light gradient boosting machine,” “categorical boosting,” “generalized additive model,” “artificial neural networks,” and “deep learning.” To maintain uniformity, we confined our search to articles published in English within the period spanning from 2004 to 2023, excluding reviews and non-original research contributions. Although reviews provide valuable syntheses and insights into the existing body of research, they do not furnish the original data that are crucial for bibliometric analysis. Bibliometric studies hinge on quantitative metrics, including citation frequencies, publication dates, and author collaboration patterns, which are predominantly extracted from original research articles. Furthermore, by excluding non-original research, we ensure that our analysis is centered on primary sources of information, thereby enhancing the accuracy and focus of our study. Following the application of these criteria, we successfully retrieved a total of 1,036 documents for subsequent analysis.

### 2.2 Bibliometric analysis and visualization

Drawing upon CiteSpace, VOSviewer, and Bibliometrix, we conducted a meticulous bibliometric review of literature data procured from the Web of Science (WOS) core database. The amassed data were systematically organized within an input folder and subsequently imported into CiteSpace 6.3R1, VOSviewer, and Bibliometrix software for comprehensive analysis. Our investigation delved into diverse aspects, encompassing country contributions, institutional affiliations, author profiles, cited authors, referenced documents, cited journals, keywords, and temporal trends spanning from 2004 to 2024. Additionally, we scrutinized the annual publication count to pinpoint research hotspots and discern emerging trends.

**Figure 1:**
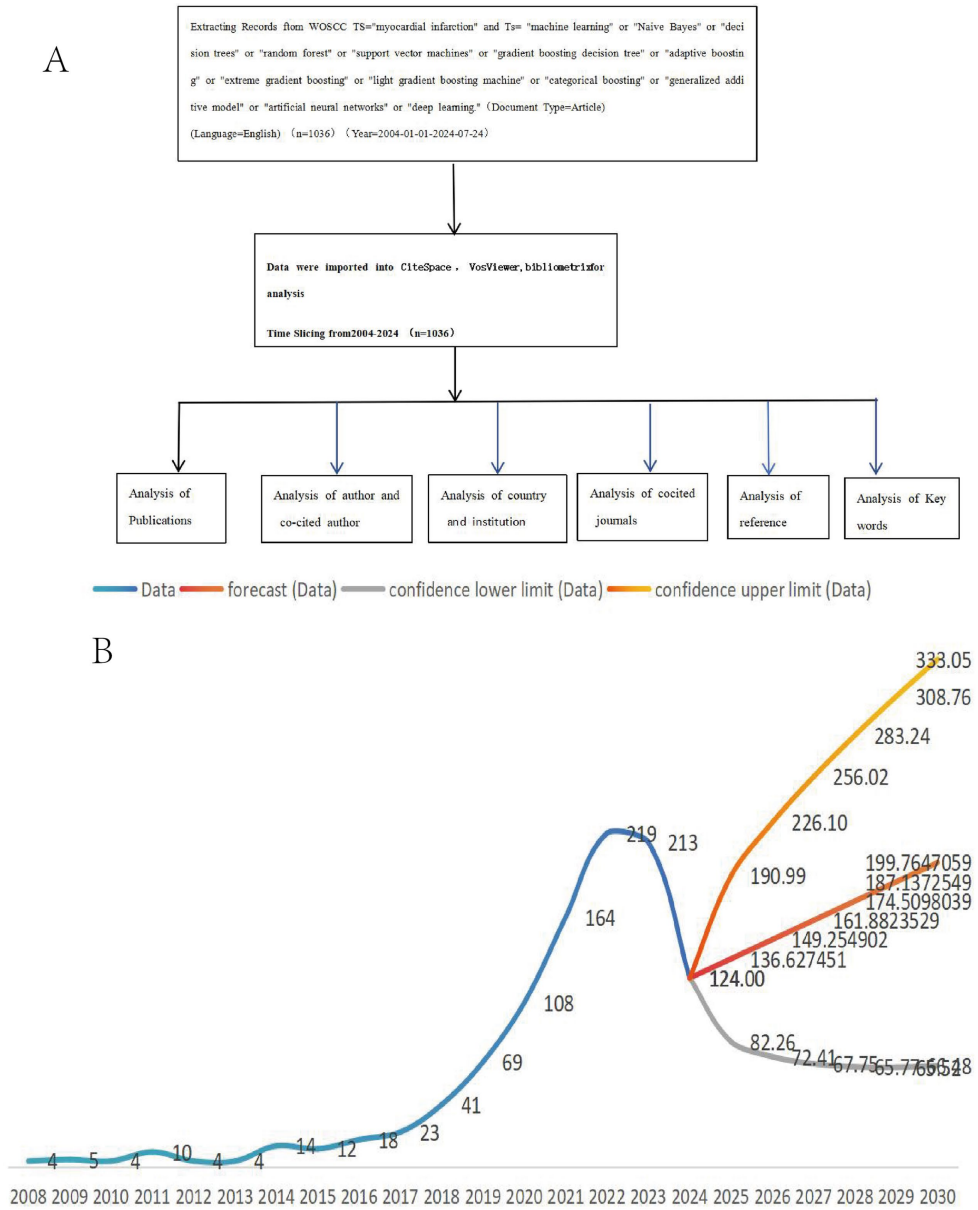
MI and ML data acquisition process and processing flow chart **(A)**. Trend diagram of publication volume in 2008–2024 MI and ML **(B)**.

Within the CiteSpace 6.3.R1 platform, we designated ‘Keyword’ as one of the node types, configuring the strength of the links with a PMI selection threshold of 0.75. Furthermore, we applied the Pathfinder algorithm for pruning selection to refine the analysis. Subsequently, the sliced networks underwent rigorous pruning, merging, and conversion processes within CiteSpace, culminating in the inclusion of 1,036 articles for final analysis. This meticulous approach encapsulates the entire data acquisition, processing, and analytical flowchart as illustrated in Figure 1A.

## 3 Results

### 3.1 Growth trend of publication

Data pertaining to the annual production of myocardial infarction (MI) and machine learning (ML) research publications were systematically compiled and analyzed using Excel software (Figure 1B). Our analysis revealed a consistently upward trajectory in the annual publication volume of MI and ML research from 2008 to 2024. In particular, the period between 2008 and 2017 witnessed a gradual yet steady increase in publications. However, 2016 emerged as a pivotal year, marking a significant global turning point where the growth rate transitioned from negative to positive, aptly designated as the ‘Year of AI’ in this context. The increased availability of medical data, advancements in machine learning (ML) algorithms, and the growing recognition of ML’s potential in healthcare all contributed to the positive growth trajectory observed after 2016. This shift underscores the escalating interest and attention garnered by ML techniques in the realm of MI research.

Subsequently, from 2018 onward, the publication volume surged at an accelerated pace, accounting for a substantial 89% of the total publications recorded during the entire study period (Figure 1B). Based on the current growth trend, we estimate that the number of publications will continue to increase, potentially surpassing 300 articles by 2030. However, this estimate is subject to uncertainties in future research funding, technological advancements, and other factors. This observation underscores the dynamic evolution and sustained relevance of this field, as researchers continue to explore innovative avenues for harnessing ML in advancing our understanding and management of myocardial infarction.

### 3.2 National and institutional analysis

#### 3.2.1 National cooperation analysis

**Figure 2:**
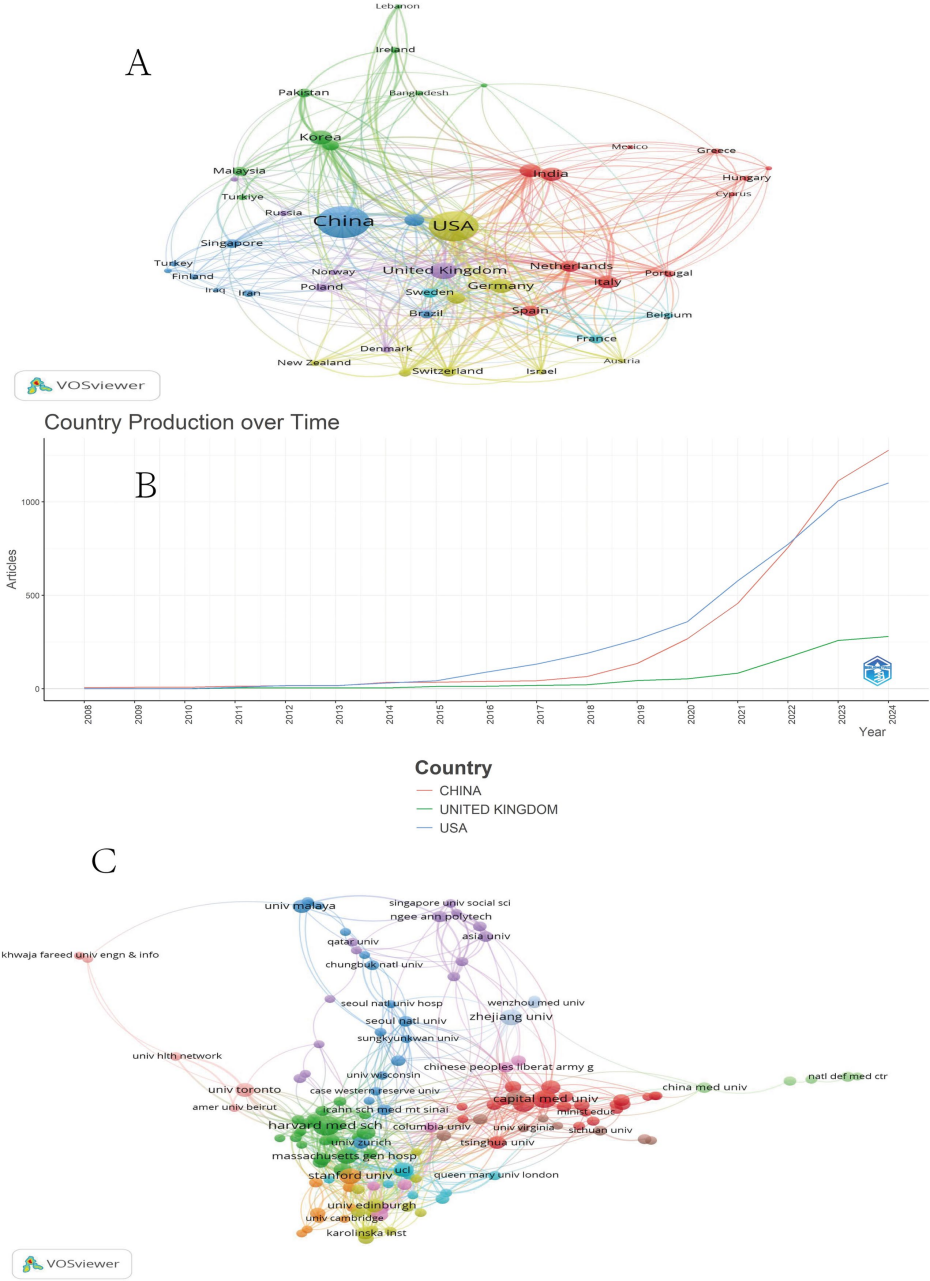
National cooperative diagram of MI and ML **(A)**. MI and ML country production over time **(B)**. MI and ML institutional cooperation **(C)**.

VOSviewer software was utilized to create the national collaboration network diagram (Figure 2A), while Bibliometrix software was employed to generate the country production diagram showcasing the top three countries in terms of publication volume (Figure 2B). In Figure 2A, countries are represented by circles, with larger circles indicating a higher publication volume. The connecting lines signify collaborations between countries, and distinct clusters are represented by different colors. The analysis of national collaboration reveals the emergence of clusters in the fields of ML and MI, with China and the United States as prominent representatives. China primarily collaborates with Asian and European countries such as Japan, South Korea, the United Kingdom, and Germany, whereas the United States mainly collaborates with Asian and European countries, including the United Kingdom, Germany, and India. Analysis of spatiotemporal development indicates that before 2015, there was a comparable level of research output among countries in MI and ML research. However, after 2015, both China and the United States experienced rapid development (a significant increase in research output and quality over a short period) in this field, starting from 2022, China’s research in this area will surpass that of the United States (Figure 2B).

#### 3.2.2 Institutional cooperation analysis

VOSviewer software was used to create the institutional collaboration network diagram (Figure 2C). In this figure, institutions are represented by circles, with larger circles indicating a higher number of publications. Different colors represent distinct clusters, while the lines connecting the circles signify collaboration between institutions. Analysis of Figure 2C reveals that in the fields of MI and ML, a cluster of institutional collaborations has formed, represented by prominent institutions such as Harvard Medical School in the United States and Capital Medical University in China. In particular, there is relatively little collaboration observed between domestic and foreign institutions.

The leading position of the United States and China can be attributed to their large scientific populations, substantial research funding, and supportive policies for healthcare innovation. To gain a more comprehensive understanding, future analyses should delve into the specific research outputs, funding allocations, and policy frameworks that have contributed to the emergence of these countries as leaders in ML and MI research.

### 3.3 Visual analysis of authors and author partnership

#### 3.3.1 Visual analysis of authors

**Figure 3:**
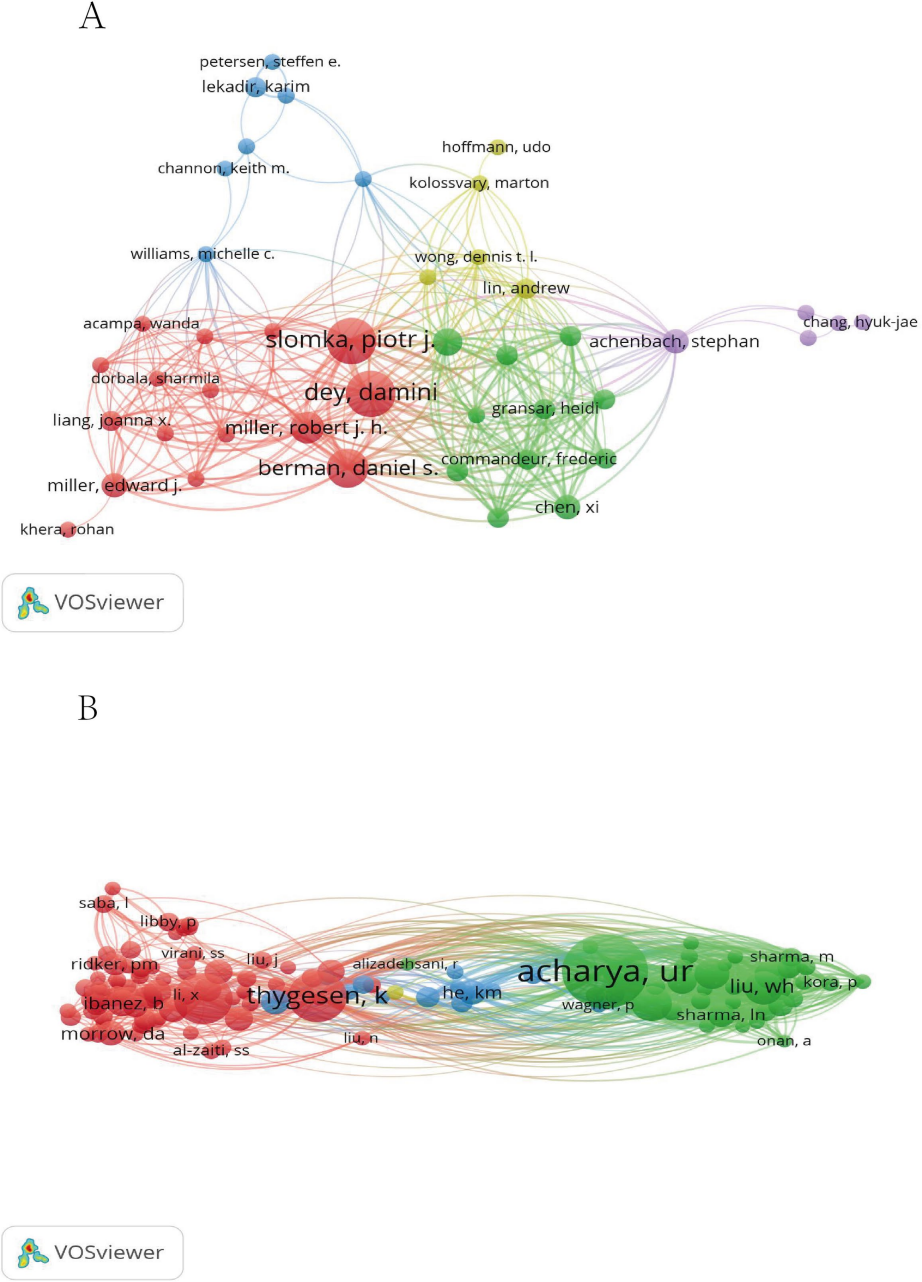
MI and ML author collaboration diagram **(A)**. MI and ML cited author collaboration chart **(B)**.

VOSviewer software was employed to generate an author collaboration network diagram (Figure 3A). Within this figure, distinct authors and their respective teams are represented by circles of varying colors, while the connecting lines depict their collaborative ties. The analysis underscores the emergence of a prominent cooperative team in the MI and ML fields, led by Dey, Damini, and Berman, Daniel S. from the United States. This team has developed and validated an advanced deep learning system capable of swiftly quantifying plaque volume and stenosis severity through CCTA. Accuracy of this system closely aligns with expert readings and intravascular ultrasound results, suggesting potential prognostic value for future myocardial infarction cases (17). This was a comprehensive multicenter study, with the research paper boasting an impact factor of 30.8 in 2023. Conversely, within the domestic landscape, collaborative author teams have yet to materialize in significant numbers. One standout figure is Chin-Sheng Lin, who has garnered attention with his four publications. Lin has developed an innovative deep learning model (DLM) that predicts biological age based on electrocardiogram data, thereby shedding light on its potential implications for future cardiovascular disease (CVD) risk assessment (18). In another study, a DLM (deep learning model) grounded on 12-lead electrocardiograms was presented as an innovative diagnostic support tool, offering a fresh perspective on clinical diagnosis (19). In 2023, the impact factors associated with these publications varied significantly, with one recording a score of 3.6, while the other had an impact factor of 0.

#### 3.3.2 The cited author analysis

VOSviewer software was utilized to generate an analysis chart showcasing cited authors (Figure 3B). In this chart, distinct clusters are represented by different colors, while the size of the circles corresponds to the number of citations. In particular, THYGESEN, K, and ACHARYA, UR exhibit the highest citation counts. ACHARYA, UR, et al., published an extensive review that delved into artificial intelligence techniques, specifically leveraging ECG and other biophysical signals for MI diagnosis. This review also highlighted the application of deep convolutional neural networks (DCNN) in MI diagnosis (11). Thygesen, K.’s seminal work, titled “Fourth Universal Definition of Myocardial Infarction (2018),” was published in the prestigious Journal of the American College of Cardiology in 2018. By 2023, this article had acquired an impact factor of 24, attesting to its significance in the field. It represents an expert consensus on myocardial infarction, encompassing contributions from esteemed scholars across various nations, including the United States, Denmark, and New Zealand (17).

### 3.4 Cited journal analysis

#### 3.4.1 Journal double-graph superposition analysis

**Figure 4:**
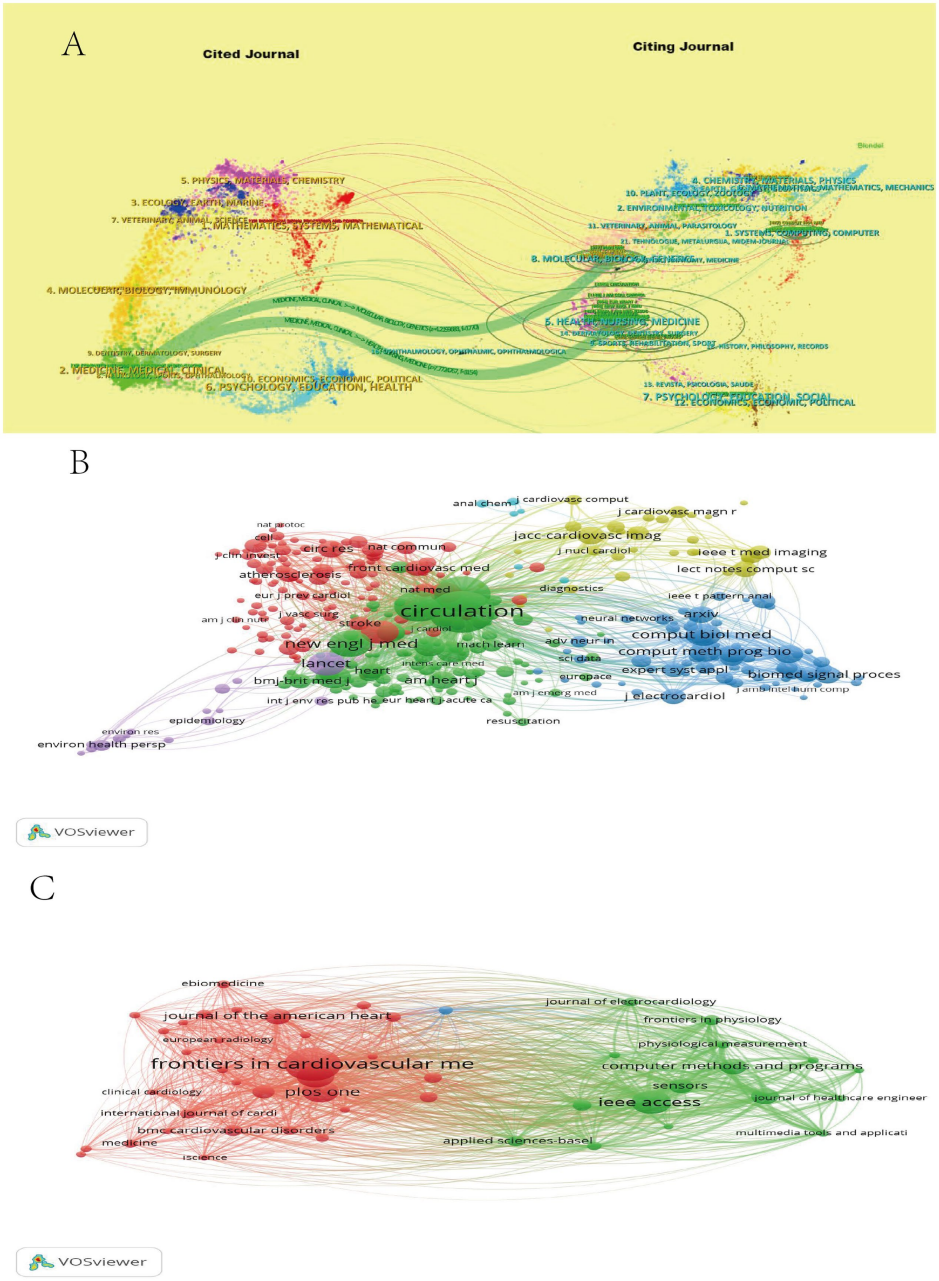
Periodicals double-map superposition of MI and ML journals **(A)**. Co-occurrence map of cited journals in MI and ML **(B)**. Journal Collaboration Map for MI and ML **(C)**.

Using CiteSpace 6.3.R1 to create an overlay of the Periodicals double-map superposition of journals (Figure 4A) allows for an understanding of research directions. This overlay presents the cited journals on the left and the citing journals on the right. Journals in the fields of Medicine, Medical Sciences, and Clinical Science have been cited by journals in Molecular Biology, Genetics (*Z* = 4.2199888), Health, Nursing, and Medical Science (*Z* = 7.7724767).

#### 3.4.2 Analysis of cited journals

VOSviewer software was utilized to generate a co-occurrence map of cited journals as depicted in Figure 4B. In this figure, journals are represented by circles, with the size of each circle indicating the number of citations received by the corresponding journal. The connecting lines between the circles represent the frequency of collaborations among the journals, while different colors denote distinct clusters. In particular, the journal with the highest number of citations is Circulation from the United States, which received 1790 citations. It is followed by the Journal of the American College of Cardiology, also from the United States, with 1,249 citations.

#### 3.4.3 Analysis of journal collaboration

VOSviewer software was utilized to generate a journal co-occurrence map as presented in Figure 4C. The analysis conducted revealed that the journal with the highest number of publications is Frontiers in Cardiovascular Medicine from Switzerland. This journal boasted an impact factor of 3.1 in 2023, with a total of 49 publications and 249 citations. On the other hand, the journal with the highest number of citations was Computers in Biology and Medicine from the United States, which garnered 538 citations, 12 publications, and had a latest impact factor of 6.7 in 2023. A comprehensive analysis underscores the prominent position occupied by Computers in Biology and Medicine from the United States in this study.

### 3.5 Cited reference analysis

The analysis of cited literature is instrumental in identifying prevalent research themes and hotspots. Utilizing CiteSpace 6.3.R1, we conducted an in-depth examination of co-cited literature, generating comprehensive tables to showcase the most influential works (Tables 1, 2). Foremost among these is the seminal work by Acharya, U. Rajendra from Ngee Ann Polytechnic in Singapore, titled “Application of deep convolutional neural network for automated detection of myocardial infarction using ECG signals.” This groundbreaking paper introduces a convolutional neural network (CNN) algorithm, revolutionizing the automated detection of myocardial infarction (MI) from ECG signals, both with and without noise contamination. Following closely in popularity is “Classification of myocardial infarction with multi-lead ECG signals and deep CNN” by Ulas Baran Baloglu from Munzur University, which showcases a CNN model tailored to multi-lead ECG analysis, achieving extraordinary accuracy and sensitivity exceeding 99% in diagnosing MI, underscoring the transformative potential of deep learning in this field (20) (Table 1). The document boasting the highest centrality is “Automated characterization and classification of coronary artery disease and myocardial infarction by decomposition of ECG signals: A comparative study,” penned by Acharya, U. Rajendra. This groundbreaking work introduces a screening system designed to empower cardiologists in the early detection of cardiovascular diseases (CAD) and myocardial infarction (MI), facilitating the precise prescription of medications and timely intervention. By leveraging advanced ECG signal analysis techniques, this study underscores the critical role of technology in improving patient outcomes and advancing the field of cardiovascular medicine (18) (Table 2).

**Table 1:** MI and ML top 10 count cited reference.

Number	Cited reference	Citation counts	Representative author (publication year)	Document type
1	Application of deep convolutional neural network for automated detection of myocardial infarction using ECG signals	56	U. Rajendra Acharya (2017)	Article
2	Classification of myocardial infarction with multi-lead ECG signals and deep CNN	49	Ulas Baran Baloglu	Article
3	Cardiologist-level arrhythmia detection and classification in ambulatory electrocardiograms using a deep neural network	33	Awni Y Hannun (2019)	Article
4	Detection of myocardial infarction in 12 lead ECG using support vector machine	29	Dohare, AK (2018)	Article
5	2017 ESC Guidelines for the management of acute myocardial infarction in patients presenting with ST-segment elevation	28	Ibanez, B (2018)	Journal Article
6	ML-ResNet: A novel network to detect and locate myocardial infarction using 12-lead ECG	26	Han, C (2020)	Article
7	A novel approach for detection of myocardial infarction from ECG signals of multiple electrodes	25	Tripathy, RK (2019)	Article
8	Heart Disease and Stroke Statistics—2018 Update: A Report From the American Heart Association	23	Benjamin, EJ (2018)	Article
9	A novel automated diagnostic system for classification of myocardial infarction ECG signals using an optimal biorthogonal filter bank	22	Sharma (2018)	Article
10	Fourth universal definition of myocardial infarction (2018)	22	Thygesen, K (2019)	Review

**Table 2:** MI and ML top 10 centrality cited reference.

Number	Cited reference	Centrality	Representative author (publication year)	Document type
1	Automated characterization and classification of coronary artery disease and myocardial infarction by decomposition of ECG signals: a comparative study	0.12	Acharya, UR (2017)	Article
2	Automated detection and localization of myocardial infarction using electrocardiogram: a comparative study of different leads	0.09	Acharya, UR (2016)	Article
3	Application of higher-order spectra for the characterization of Coronary artery disease using electrocardiogram signals	0.08	Acharya, UR (2017)	Article
4	A Novel Calibration Procedure of Pulse Transit Time based Blood Pressure measurement with Heart Rate and Respiratory Rate	0.07	Lui, Hin-Wai (2018)	Article
5	Heart Disease and Stroke Statistics—2015 Update: A Report From the American Heart Association	0.06	Mozaffarian, D	Article
6	Machine Learning and Prediction in Medicine—Beyond the Peak of Inflated Expectations	0.06	Chen, JH (2017)	Editorial Material
7	Machine Learning in Medicine	0.06	Deo, RC (2015)	Article
8	A survey on deep learning in medical image analysis	0.06	Litjens, G (2017)	Article
9	2017 ESC Guidelines for the management of acute myocardial infarction in patients presenting with ST-segment elevation	0.12	Ibanez, B (2018)	Article
10	Multiple-feature-branch convolutional neural network for myocardial infarction diagnosis using electrocardiogram	0.09	Liu, WH (2018)	Article

### 3.6 Keyword analysis

#### 3.6.1 Keyword co-occurrence and cluster analysis

**Figure 5:**
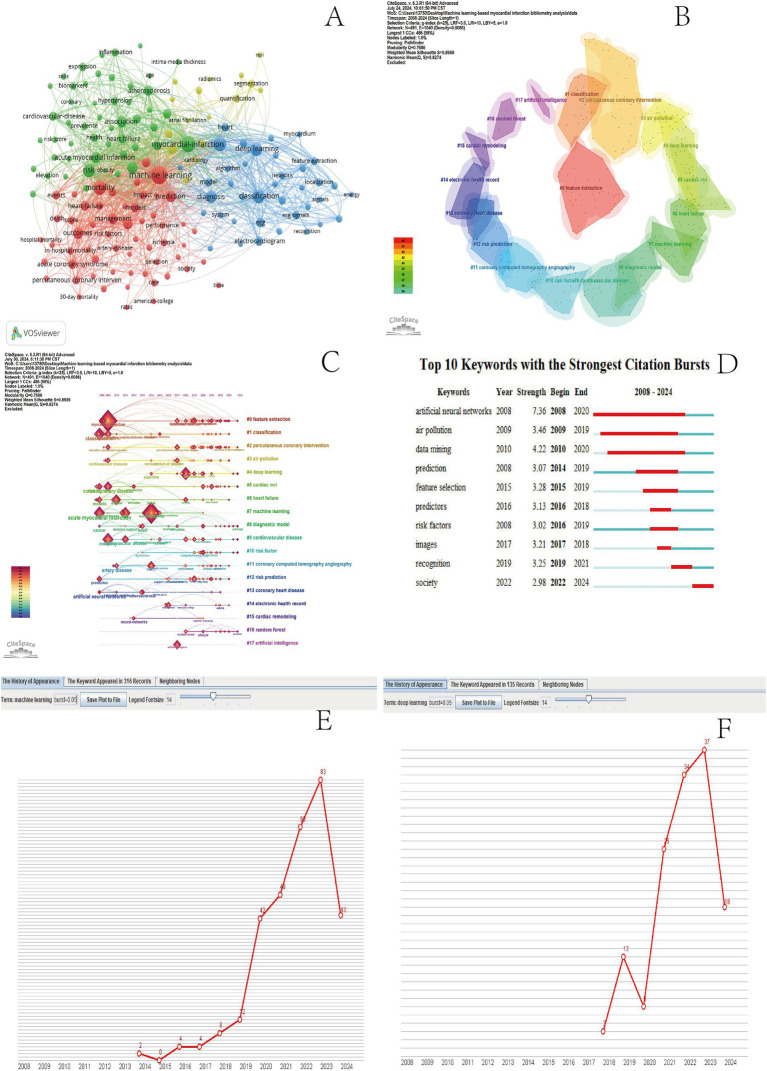
MI and ML keyword co-occurrence chart **(A)**. MI and ML keyword clustering plots **(B)**. ML and MI keywords timeline **(C)**. MI and ML Top 20 Burst keywords **(D)**. Machine learning timeline **(E)**. Deep Learning Timeline **(F)**.

Keywords in bibliometrics are the embodiment of research hot spots (19). The keyword clustering diagram generated by VOSviewer (Figure 5A) reveals three primary clusters. The first cluster revolves around “myocardial infarction,” with associated keywords such as cells, biomarkers, cardiovascular disease, inflammation, acute myocardial infarction, and risk. These keywords indicate that this cluster primarily focuses on molecular biology research. The second cluster centers on “machine learning,” with related keywords including impact, mortality, prediction, risk factors, hospital mortality, acute coronary syndrome, and in-hospital mortality. This suggests that the research within this cluster primarily adopts a clinical approach. The third cluster is centered on “Deep Learning (DL),” with associated keywords being myocardium, diagnosis, feature extraction, classification, ECG signals, electrocardiogram, and localization. This illustrates that DL research in the context of myocardial infarction primarily involves the use of machine learning techniques to extract ECG features for predicting and diagnosing risks and prognosis.

CiteSpace 6.3.R1 was employed to create an insightful keyword clustering diagram where thematic labels for each cluster were meticulously selected utilizing the log-likelihood ratio (LLR) method ensuring that each cluster’s essence is accurately captured and presented (21). A total of 21 primary clusters were extracted with the system selecting the top 18 clusters labeled as #0-#17 (Figure 5B). The modularity score of a network indicates the clarity of its structure at the level of decomposed clusters whereas the silhouette score of a cluster measures the homogeneity of its members. In this clustering the modularity score (Q) is 0.7686 and the average silhouette score (S) is 0.8959. With an average silhouette score (S) greater than 0.3 and a modularity score (Q) exceeding 0.5 this clustering is deemed credible and effective (21). The top 10 clusters in descending order are: #0 feature extraction #1 classification #2 percutaneous coronary intervention #3 air pollution #4 deep learning #5 cardiac MRI #6 heart failure #7 machine learning #8 diagnostic model and #9 cardiovascular disease (Figure 5B). These 10 clusters represent the primary research hotspots. According to the main cluster distribution table (Table 3) the oldest cluster is #1 classification. Cluster #7 machine learning has an average publication year of 2012. The youngest cluster is #16 random forest. In particular cluster #15 cardiac remodeling holds the highest silhouette score of 0.975 (Figure 5B and Table 3).

**Table 3:** Distribution map of the top 10 major clusters.

Cluster ID	Size	Silhouette	Mean (Year)	Major themes
0	50	0.835	2019	feature extraction, convolutional neural network
1	38	0.926	2012	classification, feature selection
2	37	0.856	2020	percutaneous coronary intervention, TIMI risk score
3	35	0.913	2016	air pollution, cardiovascular diseases
4	34	0.749	2019	deep learning, echocardiography
5	33	0.966	2015	cardiac MRI, generative adversarial networks
6	30	0.943	2014	heart failure, hospitalization
7	30	0.975	2012	machine learning, acute coronary syndrome
8	30	0.874	2017	diagnostic model, immune infiltration
9	29	0.917	2017	cardiovascular disease, atrial fibrillation
10	26	0.815	2019	risk factor, mitochondrial fission 4
11	24	0.924	2015	coronary computed tomography angiography, coronary plaque
12	23	0.888	2018	risk prediction, validation
13	22	0.942	2013	coronary heart disease, atherosclerosis
14	15	0.92	2018	electronic health record, ischemia
15	14	0.993	2014	cardiac remodeling, scar tissue 18
16	10	0.902	2022	random forest, macrophages
17	6	0.987	2017	artificial intelligence, subclinical

#### 3.6.2 Keywords timeline analysis

Timeline plots provide a visual representation of the duration and historical progress of each group, effectively reflecting the trends mentioned earlier (22). CiteSpace6.3.R 1 Keyword timeline map is drawn after keyword clustering (Figure 5C). A node represents a keyword, and the location of the node corresponds to the year when the keyword occurred. The size of the node is proportional to its frequency of occurrence. In the right keywords of the timeline plot cluster clusters, these clusters are labeled in different colors at different times in the lower left corner. In the keyword timeline, the keyword machine learning first appeared in 2014, and after 2018, the frequency of machine learning keywords doubled (Figure 5E). The cluster deep learning keywords first appeared in 2018, and the frequency of keyword occurrence observed a significant increase in 2021 (Figure 5F). In traditional machine learning methods, researchers usually rely on statistical learning techniques, such as support vector machines, decision trees, and random forests, to process and analyze medical data (20). These methods perform well in feature extraction and classification tasks but require substantial feature engineering work. Feature engineering is a complex and time-consuming process that requires researchers to have rich domain knowledge and experience in order to manually select and construct effective feature sets (23). Moreover, the performance of machine learning methods is often limited by the quality and quantity of selected features. With the emergence of deep learning technology, the field of mental infarction research has ushered in a major breakthrough. Deep learning, especially the convolutional neural network (CNN) and the recurrent neural network (RNN), has the ability to automatically learn and extract data features (21, 24), thus greatly reducing the dependence on feature engineering. This technique can capture complex patterns and relationships from raw medical data, in turn, providing more accurate and comprehensive disease analysis. The introduction of deep learning brings significant improvements to the risk assessment model of mental infarction. Through the training of deep neural networks, the model can more accurately identify the factors associated with the risk of infarction of patients and even predict the patients’ disease progression. Moreover, in addition to risk assessment, deep learning has also played a key role in the diagnosis and treatment strategies of myocardial infarction. For example, in medical imaging analysis, deep learning techniques can accurately identify and segment abnormal regions in cardiac imaging (25) to provide doctors with a more accurate basis for their diagnosis. At the same time, through deep mining of patients’ medical history and physiological data, deep learning helps developing more personalized and effective treatment plans (26). The transition from machine learning to deep learning marks a new era for the study of myocardial infarction. Deep learning not only improves the efficiency and accuracy of data processing but also provides more powerful and accurate tools for the prevention, diagnosis, and treatment of myocardial infarction (2) with the continuous progress of technology and the increasing wealth of medical data, it is reasonable to believe that deep learning will play an increasingly important role in the field of mental infarction research. According to the timeline analysis of keywords and clusters, the latest study is # 16 random deep forest. The latest study shows that the machine learning method of random forest is applied to establish a reliable diagnostic prediction and diagnostic model for the diagnosis of myocardial infarction, which provides potential genetic markers for the screening of early-stage disease (27, 28).

#### 3.6.3 Keyword bursts and timeline analysis

Keyword outbreak refers to keywords that are used more frequently in a short period of time and can predict research trends and research frontiers in the field (29). CiteSpace6.3.R1 Draw the keyword bursts map (Figure 5D). Red figure represents the outbreak time of the keyword, indicating that the keyword is frequently cited during this period, and the blue line represents the appearance and duration of the keyword. The keyword with the longest and strongest outbreak time in 2008–2020 is artificial neural networks (ANN), indicating that the application of ANN in MI and ML research fields is the research trend. The latest outbreak keyword of 2022–2024 is society. According to the relevant literature analysis, the deep learning system can predict mechanical infarction by rapidly measuring plaque volume and stenosis severity by coronary computed tomography angiography (CCTA) (17, 26) is the latest research trend in the field of MI and ML.

## 4 Discussion

Since the emergence of research literature on MI and machine learning (ML) in 2008, interest in this area has grown rapidly, especially since the pivotal moment in 2016. The number of articles published in 2016 was 18, and 219 in 2022 was 12 times that published in 2016. In the fields of ML and MI, China and the United States are leading representatives. Since 2015, both China and the United States have witnessed rapid development in these research areas. However, in terms of research quality, the United States significantly surpasses China. Institutional collaborations have formed, represented by partnerships such as Harvard Medical School in the United States and Capital Medical University in China. However, there is still a need to strengthen cooperation among domestic and foreign institutions. This shows that both the United States and China have a large scientific population and sufficient financial resources, which have had a huge impact on medical scientific research, highlighting their dominant position in the field of medical scientific research. The reasons for the lack of cooperation between Chinese and American institutions are mainly the national financial institutions and investment environment, cultural differences, and so on. In the field of MI and ML research, collaborative teams have emerged, represented by Dey, Damini, and Berman, Daniel S from the United States. Chinese scholars need to enhance their cooperation, focusing on both qualitative and quantitative development. Overall, the research areas of MI and ML are oriented toward Medicine, Medical Science, Molecular Biology, and Genetics. Circulation and Computers in Biology and Medicine from the United States occupy an important position in this study.

The application of ML in myocardial infarction (MI), especially acute myocardial infarction (AMI), and the characteristic application of ML was explored in this study. Early warning and prognostic management of recurrent AMI are current research challenges. This is compounded by the increasing trend of young individuals leading chaotic lifestyles and consuming unhealthy diets (30). Early diagnosis and intervention in cases of MI can greatly enhance the prognosis for patients with AMI. Presently, the evaluation of cardiac biomarkers such as cardiac troponin I and cardiac troponin T is regarded as a key method for diagnosing AMI; nevertheless, the diagnostic accuracy of AMI using these biomarkers remains suboptimal due to their limited sensitivity and specificity. ML technology automatically identifies early risk markers for sudden cardiac death on measurable arrhythmia parameters (27, 31). Deep learning is a form of ML that involves automated extraction and selection processes. It is widely used for its convenience, high performance, and capability to handle large datasets.

### 4.1 The MI and ML bibliometric analysis mainly draws the following three conclusions

#### 4.1.1 Deep learning is an emerging research direction in MI

Myocardial infarction (MI), as one of the leading causes of death and disability worldwide, requires early and accurate diagnosis for timely intervention and improved patient outcomes. Traditional electrocardiogram (ECG) analysis relies on the experience and subjective judgment of doctors, posing risks of misdiagnosis and missed diagnosis. The introduction of deep learning technology provides a new solution for automatic, rapid, and accurate diagnosis of MI.

Deep learning is very promising in extracting cardiac strain information quickly and accurately (4, 32). Deep learning will be accelerating from 2021. Deep learning in a wide range of visual applications shows a strong ability, to learn effective hierarchy, all kinds of deep learning architecture (such as deep neural networks, deep belief networks, and recurrent neural networks) have been widely used in the medical field, the results are comparable to human experts, even in some cases is superior to human experts (33). One of the advantages of deep learning models is that they can achieve high-precision performance without a large amount of signal processing, and usually, only a small number of signals are needed to effectively classify signals. In recent studies, none of the authors who developed deep models for MI detection explained how these models made predictions, and the lack of interpretation of these model mechanisms also poses challenges, making clinicians lose confidence in using deep models to assist diagnostic decisions in clinical settings (4). In recent years, the application of deep learning in MI research has been emerging. For example, a CNN-based automated ECG analysis system can detect ST-segment elevation myocardial infarction (STEMI) in real time to win valuable time for emergency treatment (34), The LSTM model achieves early warning and risk assessment of MI by continuously monitoring ECG signals in patients (35). In addition, there are studies applying deep learning to cardiac MRI image analysis to help doctors identify myocardial scar tissue after myocardial infarction (36).

### 4.2 Neural networks based on deep learning frameworks are a research hotspot in MI studies

With the rapid development of artificial intelligence technology, deep learning, as a branch of machine learning, is increasingly widely used in the medical field. Especially in the study of myocardial infarction (MI), neural networks based on deep learning framework show great potential (37).

### 4.3 Application of deep learning in early diagnosis of MI

Among ML techniques for predicting coronary heart disease (CHD), neural networks (NN) are widely used to improve performance accuracy (38). In recent years, convolutional neural network (CNN), as a deep learning method, has been widely used in many fields such as medical image analysis (39). Attallah’s team proposed Auto-MyIn, an automatic diagnostic tool that utilizes multiple convolutional neural networks (CNNs) to diagnose myocardial infarction (MI) (40).

Neural networks under the deep learning framework, such as convolutional neural network (CNN), recurrent neural network (RNN), and its variant long short-term memory network (LSTM), have been successfully applied to the analysis of electrocardiogram (ECG) signals to achieve early diagnosis of MI, These networks can automatically extract complex features from the original ECG signals; without manual intervention, they significantly improved the diagnostic accuracy and efficiency (41). In addition, combined with transfer learning technology, the deep learning model can also perform well on small sample data sets, further promoting its application in clinical practice (42). In the future, we can use six-layer random pooling convolutional neural network and multichannel data (43) to enhance the prediction of myocardial infarction.

### 4.4 Application of deep learning in MI risk assessment

Apart from early diagnosis, deep learning also plays a crucial role in MI (myocardial infarction) risk assessment (37). By integrating multisource heterogeneous data such as patients’ medical history, physiological data, genetic information, and imaging data, deep learning models can construct a more comprehensive risk assessment system (44). These models can capture subtle differences that are difficult to identify with traditional methods, providing patients with more personalized risk predictions and assisting doctors in formulating more precise treatment plans.

### 4.5 Application of deep learning in MI rehabilitation therapy

Deep learning also shows great potential in the MI rehabilitation phase. By analyzing the rehabilitation process data, the deep learning model can monitor the patient’s recovery status in real time and adjust the rehabilitation plan according to the patient’s specific situation. In addition, deep learning can combine virtual reality (VR) and augmented reality (AR) technology (45) to provide patients with a more immersive rehabilitation experience and improve patients’ compliance and rehabilitation effect.

### 4.6 Challenges and prospects

Despite the remarkable advances in MI research, deep learning still faces many challenges. First, the acquisition and annotation of medical data are expensive, which limits the large-scale application of deep learning models. Currently, regardless of which machine learning model is used to detect myocardial infarction (MI) depends on ECG detection. With its reasonable price, the ease of implementation in clinics, and the advantages of immediate access to test results. Although ECG machines are easy to deploy in both rural and urban areas, however, an accurate diagnosis of cardiac disease using an ECG still needs to be performed by adequately trained cardiologists. Unfortunately, rural areas may not always be staffed with such professional qualifications (46). Future research should focus on addressing these issues, while exploring the integrated applications of deep learning with other emerging technologies, such as generative adversarial networks (GAN) (47), to further improve the effectiveness and value of deep learning in MI research.

The second direction is exploring the application of machine learning in myocardial infarction (MI) rehabilitation therapy. Rehabilitation therapy after MI is crucial for patient recovery. By utilizing machine learning techniques, we can develop more personalized rehabilitation treatment plans based on the specific conditions of each patient, thereby enhancing their recovery outcomes and quality of life. For example, using convolutional neural networks (CNN) to detect MI signals for urban healthcare in smart cities has achieved an accuracy rate of 99.22%, a sensitivity of 99.15%, and a specificity of 99% (48). It has been reported that visual deformers can replace CNN in the medical field (49). Future research on the application of visual deformers in the field of myocardial infarction is also possible. Studies have reported that the U-net based on the Bayesian transformer is used to separate cardiac structures from T1 mapping images, which can significantly distinguish healthy people from myocardial infarction and other myocardial infarction, distinguish between diseases (50). The third direction is to investigate how machine learning can assist in the early diagnosis of cardiovascular diseases (51). The identification of early symptoms of cardiovascular diseases is of great significance for timely treatment and preventing the worsening of the condition (52).

The fourth direction is to develop an intelligent cardiovascular disease management system based on machine learning (53). By integrating various physiological data, medical history information, and treatment feedback from patients, we can construct an system based on machine learning (37).

## 5 Strengths and limitations of this study

This study utilized CiteSpace 6.3.R1 software to analyze global research trends in MI and ML. Excel, CiteSpace 6.3.R1, and other statistical tools were employed to calculate bibliometric characteristics such as publication numbers, countries, institutions, authors, cited authors, cited documents, cited journals, and keywords. It is important to note that this study is constrained to the bibliometric analysis of documents sourced from the WoS database, which may introduce bias into the data, leading to an incomplete sample size for data analysis. Other database searches should be added in future studies to obtain comprehensive data information. The CiteSpace analysis itself has some limitations, such as the inability to distinguish between the corresponding author and the first author.

## 6 Conclusion

Since 2016, machine learning and myocardial infarction research have emerged as prominent areas of study, with the United States leading in contributions. The utilization of deep learning and artificial neural networks in the context of acute myocardial infarction has become a key research focus. Challenges remain when detecting myocardial infarction by using deep learning algorithms (54). Automated systems bring potential improvements to the automated detection and localization of myocardial infarction in clinical practice (55). The development of a single microcontroller system based on the edge computing paradigm for automatic detection of myocardial infarction (56) will be a future research direction. This system is capable of real-time monitoring of patients’ health status, providing personalized treatment recommendations and lifestyle guidance, and ultimately helping patients better manage their cardiovascular diseases. Extreme learning machine (ELM) is a training algorithm for a single hidden layer forward neural network (SLFN), which converges much faster than traditional methods (57), whether there are advantages to applying this algorithm to myocardial infarction requires further validation.

In summary, future research on MI and ML will focus on improving the prediction accuracy of myocardial infarction risk, optimizing rehabilitation therapy, assisting early diagnosis, and developing intelligent cardiovascular disease management systems (58, 59). For example, a new deep learning method is used to enhance the prediction of incident cardiomyopathy by combining metabolomics with clinical risk factors (60). These studies may help us better understand the pathogenesis of cardiovascular diseases, enhance treatment outcomes, and improve patients’ quality of life.

## Data Availability

The original contributions presented in this study are included in this article, further inquiries can be directed to the corresponding author.
